# Drivers and Barriers of Wine Consumption Among Predominantly Young, Highly Educated Chinese Consumers: A Sociodemographic and Network Analysis

**DOI:** 10.3390/foods15132253

**Published:** 2026-06-23

**Authors:** Lin Zhu, Xinshu Jiang, Yulin Fang, Xiangyu Sun

**Affiliations:** 1 College of Enology, Northwest A&F University, Yangling 712100, China; xnzlin@nwafu.edu.cn (L.Z.); fangyulin@nwsuaf.edu.cn (Y.F.); 2 College of Tourism and Management, Shaanxi A&F Technology University, Yangling 712100, China; 3 The Graduate School of Technology and Innovation Management, Hanyang University, Seoul 04763, Republic of Korea

**Keywords:** wine consumption, network analysis, Gaussian graphical model, consumer behavior

## Abstract

Understanding the drivers and barriers of wine consumption is of substantial importance for both market development and sensory science research, and this is particularly salient in rapidly changing non-Western markets. Young, highly educated Chinese consumers represent one of the fastest-growing segments in the global wine market, yet large-scale studies of their consumption preferences and rejection patterns remain limited. This study aimed to characterize the conditional dependence structure of wine-consumption behavior in this population and to examine the associations between common consumption barriers and sociodemographic variables. A nationwide cross-sectional online survey collected 4823 valid responses. Non-parametric tests were used to compare sociodemographic groups, and a regularized Gaussian graphical model (GGM) was estimated to characterize the conditional associations among 15 consumption-behavior variables. The sample was dominated by young respondents (18–24 years) and individuals with higher education. The three most frequently endorsed barriers were taste aversion (51.1%), price sensitivity (38.7%), and lack of knowledge (19.6%). Age and education were the most central sociodemographic variables in the network. The knowledge barrier showed a moderate negative conditional association with education (partial *r* ≈ −0.171), whereas taste aversion—although the most frequently endorsed barrier—did not show clear conditional associations with sociodemographic variables in the network. Gender was not conditionally associated with any other variable in the network. These observations suggest that the three consumption barriers may operate through different network pathways and may therefore have different implications for intervention design, a possibility that warrants further confirmatory and longitudinal research.

## 1. Introduction

Among all alcoholic beverages, wine occupies a unique position, combining agricultural, cultural, and symbolic attributes [1]. Although wine accounts for only about 8% of global alcoholic beverage consumption by volume [2], its influence on sales value far exceeds its share of volume because of the premiumization trend [3]. While per capita consumption is declining in traditional European markets (e.g., Italy, France, Spain), emerging markets are growing rapidly [4]. In particular, China is projected to surpass the United States and France to become the world’s largest wine-consuming market by 2030 [5]. The principal driving force behind this growth is the young, highly educated urban population, whose consumption preferences have shifted from traditional status-driven motives toward esthetic and personal-enjoyment-driven motives [6,7,8].

Understanding the behavioral drivers and barriers of wine consumption in emerging markets is therefore of considerable theoretical and practical importance. From a theoretical perspective, wine-consumption research involves the integration and analysis of multiple factors—price, brand, region of origin, grape variety, awards, and consumer segmentation—and the complexity arising from this multifactor interplay makes it a distinctive case [1]. From a practical perspective, the evolution of the Chinese wine market will affect global supply chains, agricultural planning in wine-producing regions worldwide, and public health policy. However, despite the significance of this topic, behavioral research on young Chinese wine consumers remains far less developed than research on equivalent populations in Mediterranean Europe, North America, or Australia [9,10].

Previous consumer-behavior research has identified several sociodemographic variables that robustly predict wine-consumption involvement. Age has consistently been one of the strongest predictors: Older respondents tend to report higher consumption frequency and a more pronounced preference for traditional wine styles (e.g., aged red wines) [11,12,13], together with a higher willingness to pay [14]. The relationship with education is more complex—some studies find that more highly educated individuals possess richer product-category knowledge [15,16] and greater subjective confidence in wine choice [17,18]; the relationship with overall alcohol consumption is often positive or mixed rather than consistently negative. Gender effects also vary markedly across cohorts: traditional studies typically report substantially higher consumption among men than women [19,20], whereas this gender gap has narrowed considerably in younger and more highly educated populations, with a consistent trend observed worldwide [9,21,22].

The literature identifies social facilitation, cultural and esthetic appeal, and perceived health benefits as principal motivations for wine consumption [7,23,24,25], with consumption structured by occasions such as business banquets, family meals, and solo drinking [13,26,27]. Recurrent barriers are sensory dislike of wine [23,24,28,29], price [14,23,24], and lack of knowledge or confidence [15,16,18]. Barriers are typically reported as endorsement frequencies, and frequency has often been read directly as an indicator of importance for market development—an inference that conflates the prevalence of a barrier with its structural role in consumption behavior, and which the present study examines explicitly.

Beyond these aggregate growth projections, the Chinese market has distinctive structural features: wine is deeply embedded in gift, banquet, and ‘face’ culture [8,26], red wine has historically dominated the category, and premiumization has been rapid [3]. Research on Chinese consumers, however, remains fragmented: existing studies are often regional (e.g., Beijing [10]), based on modest samples, and analyzed with univariate or regression methods [30,31], leaving the joint dependence structure of behaviors, motives, and barriers uncharacterized.

Current research on wine consumer behavior in emerging Asian markets has largely relied on univariate analytic approaches—for example, frequency descriptions, pairwise chi-square tests, and regression models that include multiple sociodemographic and attitudinal variables as mutually independent covariates [32]. Although these methods have provided a useful cumulative body of evidence, they have an inherent limitation when dealing with complex behavioral systems: They treat each predictor as an isolated entity and fail to model the conditional dependence structure among the predictors themselves [32,33]. To simultaneously characterize the conditional dependence structure among multiple consumption-behavior variables, we adopted a regularized partial-correlation network approach [32]. This approach has recently been applied to alcohol consumption research in a Spanish population [9]. For multifactor consumption systems—where demographics, attitudes, motives, occasions, and barriers are mutually entangled—this conditional-dependence view complements regression by distinguishing direct from indirect associations and by identifying structurally central variables. No large-scale network characterization of wine-consumption behavior in an emerging Asian market has yet been reported; the present study addresses this gap.

The present study addresses these research gaps through a nationwide cross-sectional survey of predominantly young, highly educated Chinese adults using a GGM. Specifically, this study has three interrelated objectives: (i) to characterize the conditional dependence structure among sociodemographic variables, overall wine-consumption involvement, consumption occasions, consumption motivations, and consumption barriers; (ii) to identify the most central nodes in this network; and (iii) to examine the relationship between descriptive frequency and structural centrality for the three consumption barriers. By integrating sociodemographic variables and behavioral characteristics, this study aims to clarify the hierarchy of drivers that promote wine consumption and the underlying mechanisms that hinder its uptake, thereby providing scientific evidence for understanding the complex drinking patterns of young Chinese consumers [25,31].

Accordingly, three research questions guided the analysis. RQ1: What is the conditional dependence structure among sociodemographic characteristics, overall consumption involvement, consumption occasions, motivations, and barriers in this population? RQ2: Which variables occupy the most central positions in this structure? RQ3: Do the three consumption barriers differ in their network embedding, and does structural centrality track descriptive endorsement frequency?

This study makes three contributions. First, it provides a large-scale network-analytic characterization of wine-consumption behavior in an emerging market (*N* = 4823), focusing on the segment most often credited with driving the future growth of the Chinese market. Second, methodologically, it extends regularized partial-correlation network analysis—mainly applied in health and psychopathology research to date and only recently to beverage consumption [9,32]—to wine-consumer research, complementing the univariate and regression-based evidence that currently dominates the field. Third, substantively, it documents a dissociation between how frequently a consumption barrier is endorsed and how strongly it is conditionally connected to other variables, a distinction with direct implications for the design and targeting of market and educational interventions.

## 2. Materials and Methods

### 2.1. Study Design and Sampling

This study used a cross-sectional quantitative design to examine wine-consumption behavior among Chinese adult consumers. The target population was adults aged 18 years or older who are currently residing in mainland China. Respondents who self-reported a chronic condition that contraindicated alcohol consumption (e.g., liver disease, pregnancy) were excluded from the analytic sample.

Data were collected from December 2024 to April 2025 through a snowball non-probability convenience sampling strategy; the questionnaire was hosted on the SoJump platform. To maximize sociodemographic diversity, the questionnaire was distributed through both online and offline channels. Online questionnaires were disseminated via social media and institutional mailing lists. In parallel, the questionnaire was distributed at wine retail stores, tasting events, and selected restaurants to reach respondents with lower online participation. This snowball sampling strategy followed the approach of Sandri et al. [10], who recruited respondents through social media networks, supplemented here with offline channels to enhance sample diversity. Upon completion, respondents could enter a platform-administered lottery for a small monetary reward; embedded attention checks and completion-time screening were used to mitigate any incentive-induced careless responding [34].

The sample size was determined a priori based on two considerations: (i) the requirements of network analysis—stable edge-weight estimation requires at least 10–20 observations per parameter to be estimated [32], and with 15 candidate nodes and up to 105 potential edges in the present study, the minimum required sample size was approximately 1050–2100; (ii) the feasibility of subgroup analyses. After excluding invalid questionnaires, a valid sample of *N* = 4823 was retained.

### 2.2. Ethical Considerations

This study was conducted in accordance with the principles of the Declaration of Helsinki and was approved by the Institutional Review Board (IRB) of Northwest A&F University (approval no.: 2025-WYHC-0013; approval date: 26 December 2024). All respondents provided electronic informed consent (online version) before completing the questionnaire. No personally identifiable information was collected during the study.

### 2.3. Instruments and Measures

The instrument used in this study was a structured self-administered questionnaire comprising 10 items spanning six conceptual domains: sociodemographic characteristics, overall wine-consumption involvement, consumption occasions, consumption motivations, consumption barriers, and country-of-origin preferences. The item pool was developed by drawing on established frameworks in wine-consumer-behavior research [1] and was then adapted to the Chinese context through expert consultation: five wine-industry practitioners and two consumer-behavior researchers reviewed the items for face and content validity and suggested refinements to wording and response options prior to deployment. A pilot study with 52 respondents confirmed comprehensibility and indicated a median completion time of approximately 3 min.

#### 2.3.1. Sociodemographic Variables

This study measured four sociodemographic variables: age (Q1; five-category ordinal: 18–24, 25–34, 35–44, 45–54, and 55+); gender (Q2; binary: male = 0, female = 1); education (Q3; four-category ordinal: high school or below, junior college, bachelor’s, master’s or above); and city tier (Q4; four-category ordinal reflecting the level of urban development).

#### 2.3.2. Wine-Consumption Behavior

Overall wine-consumption involvement was measured through three single-choice ordinal items: overall attitude toward wine (Q5), which asked “Overall, what is your attitude toward wine?” with three response options coded 1 = negative (Dislike), 2 = neutral (Neutral), and 3 = positive (Like)—the percentage with a positive attitude reported in Section 3.2 is the proportion selecting the positive option, with the full distribution being positive 67.9%, neutral 24.6%, and negative 7.5%; price tolerance per bottle (Q6; five categories, from ≤100 CNY to >500 CNY); and annual purchase frequency (Q7; four categories, from almost no purchase to >12 bottles).

#### 2.3.3. Multiple-Choice Domains

Three items used a multiple-choice response format. Prior to statistical analysis, each multiple-choice item was decomposed into several binary indicators—one per option—following standard practice [32]. Unchecked options were coded as 0, denoting non-endorsement; this convention was made explicit to respondents through the item instructions. The complete list of variable operationalizations—including coding scheme, endorsement rate, and decision on network inclusion—is presented in Table 1.

The instrument was designed as a brief behavioral inventory rather than a multi-item psychometric scale: single-choice constructs were each measured by one ordinal item, and the multiple-choice domains were check-all-that-apply checklists whose options denote alternative behaviors rather than parallel indicators of a latent trait. Internal-consistency coefficients and factor-analytic procedures, which presuppose multiple reflective indicators per construct, are therefore not applicable to this instrument; this single-indicator approach is consistent with the network-analytic framework, in which observed variables are modeled as distinct interacting entities [33], and parallels the instrument design of Sandri et al. [9].

### 2.4. Data Preprocessing

Raw questionnaire data were exported as UTF-8-encoded CSV files. Data cleaning followed a six-step protocol: removing duplicate responses based on IP address and browser fingerprint; removing questionnaires with a total completion time of less than 60 s, which were considered indicative of possible careless responding [34]; removing questionnaires that failed any embedded attention checks; coding single-choice items as ordinal numerical variables and decomposing multiple-choice items into separate binary indicators; inspecting variable distributions and removing binary variables with endorsement rates below 5% or above 95%; and performing listwise deletion for cases with missing values on the final set of analytic variables. After these steps, a complete analytic sample of *N* = 4823 was obtained.

Three variables were excluded from the network model, each for an explicit reason. Business occasion (Q8d) was endorsed by only 2.7% of respondents, below the 5% threshold under which tetrachoric correlations become unstable [32]. Taste attraction (Q9d) is conceptually a near-mirror of taste aversion (Q10a); retaining both would have introduced a redundant, content-overlapping dyad whose strong association is definitional rather than substantive. Geographic location (Q4) was excluded for both methodological and conceptual reasons. The Gaussian graphical model is estimated from a polychoric/tetrachoric correlation matrix, which requires ordinal or binary indicators underlain by a continuous latent variable; as originally collected, geographic location is a nominal variable (province/city) and is therefore not suitable for inclusion. Although it could in principle be recoded into an ordinal city-tier variable, doing so would introduce additional measurement uncertainty for two reasons: location was obtained through an optional automatic geolocation feature, so for respondents who enabled it, the recorded position may not correspond to their actual place of residence, and the mapping from province/city to tier depends on the classification scheme adopted, which is partly subjective. Moreover, city tier is a macro-level geographic construct, whereas the remaining variables are individual-level psychological and behavioral measures; combining the two levels within a single individual-level partial-correlation network is conceptually less clean. Geographic information was therefore used only to describe the sample (Table 2).

### 2.5. Statistical Analysis

All statistical analyses were conducted in R version 4.3 using the bootnet (v1.5.6), qgraph (v1.9.8), and psych (v2.4.1) packages. The level of statistical significance was set at *α* = 0.05.

#### 2.5.1. Descriptive Statistics and Sociodemographic Group Comparisons

Descriptive statistics were computed for all variables. The Shapiro–Wilk test indicated that the distributions of all ordinal variables departed from normality (*p* < 0.001); consequently, all comparisons were performed using non-parametric tests. Comparisons of ordinal variables between two groups used the Mann–Whitney U test, with effect sizes reported as the rank-biserial correlation coefficient (*r*). Comparisons across multiple groups used the Kruskal–Wallis *H* test, with effect sizes reported as *η*^2^ and classified according to Cohen’s criteria [35]. Associations between two categorical variables were assessed with Pearson’s chi-square test, with effect sizes reported as Cramér’s *V*. Four subgroup contrasts were specified a priori on the basis of the literature reviewed in Section 1: (i) price tolerance across age groups [12,14]; (ii) knowledge-barrier endorsement across education levels [15,16]; (iii) overall attitude between genders [20,22]; and (iv) consumption occasions across age groups [13,27]. These contrasts are reported in Section 3.3.1, Section 3.3.2, Section 3.3.3 and Section 3.3.4.

#### 2.5.2. Regularized Gaussian Graphical Model (GGM)

To examine the conditional dependence structure among consumption-behavior variables, this study estimated a regularized partial-correlation network following the method of Epskamp and Fried [32]. The input to the GGM was a polychoric correlation matrix among the 15 variables; this approach is appropriate for networks composed of ordinal and binary variables, as it estimates correlations among the underlying continuous distributions [32]. The network was estimated using the graphical LASSO algorithm, with the regularization parameter *λ* selected via the Extended Bayesian Information Criterion (EBIC); the hyperparameter *γ* was set to 0.5 [36], the recommended default for exploratory network analysis. After regularization, partial correlations with an absolute value below 0.03 were set to zero in the visualization to ensure graphical clarity. With *N* = 4823 respondents and 15 candidate nodes, the observation-to-parameter ratio was 4823/105 ≈ 46, substantially above the recommended minimum ratio of 10–20 for stable GGM estimation suggested by Epskamp et al. [32].

Three considerations motivated the choice of a regularized GGM over conventional alternatives. First, multiple regression designates a single variable as the outcome and treats the remaining variables as mutually independent covariates; it therefore cannot characterize the conditional dependence structure among the explanatory variables themselves, which is the focus of the present study [32,33]. Second, structural equation modeling presupposes an a priori measurement model and directional paths; because the present instrument comprises single-item behavioral indicators rather than validated multi-item reflective scales, specifying latent constructs and directional hypotheses would be premature at this exploratory stage. Third, the regularized GGM provides a data-driven map of unique pairwise associations in which every edge is adjusted for all other variables in the model, directly serving the descriptive aim of this study [32]. The approach also has limitations: edges are undirected and carry no causal interpretation; LASSO regularization deliberately biases estimates toward sparsity and may suppress weak true associations; and the estimated structure is conditional on the set of variables included in the model [32,33,37].

This modeling choice for mixed ordinal and binary data also has implications that should be made explicit [38]. The model presupposes approximately normally distributed latent liabilities underlying the ordered-categorical responses. Tetrachoric estimates can be unstable for items with extreme endorsement rates; items endorsed by fewer than 5% or more than 95% of respondents were therefore excluded (Section 2.4), and the large sample (*N* = 4823) further stabilizes the estimation. Finally, regularized estimation on a polychoric input may retain occasional spurious small edges; the bootstrap analyses reported in Section 3.6 and the tentative interpretation applied to edges below |partial *r*| = 0.10 address this risk [32,38].

#### 2.5.3. Centrality Index Computation

Two centrality indices were computed: node strength, defined as the sum of the absolute values of all edges connected to a node; and expected influence (EI), defined as the signed sum of all edges connected to a node [39]. Following the recommendation of Bringmann et al. [40], closeness and betweenness centralities were not interpreted because of their limited reliability in sparse regularized networks.

#### 2.5.4. Network Accuracy and Stability Assessment

Following Epskamp et al. [37], the accuracy and stability of the network were assessed with the bootnet (v1.5.6) package. Edge-weight accuracy was evaluated with a nonparametric bootstrap (1000 resamples), from which 95% bootstrapped confidence intervals and edge-difference tests were obtained. The stability of centrality indices was evaluated with a case-dropping subset bootstrap and quantified by the correlation-stability (CS) coefficient, i.e., the maximum proportion of cases that can be dropped while retaining, with 95% certainty, a correlation of at least 0.70 with the original centrality order; CS values above 0.25 are considered acceptable and above 0.50 preferable [37].

## 3. Results

### 3.1. Sample Sociodemographic Profiles

**Figure 1:**
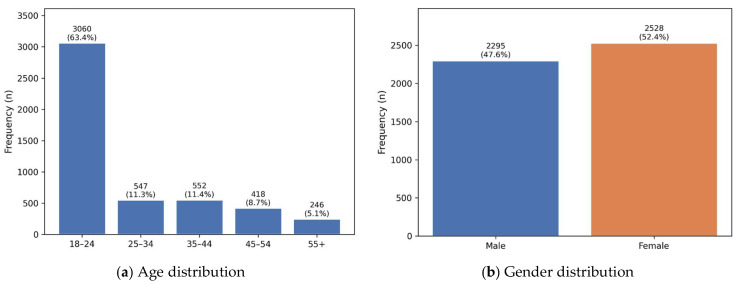

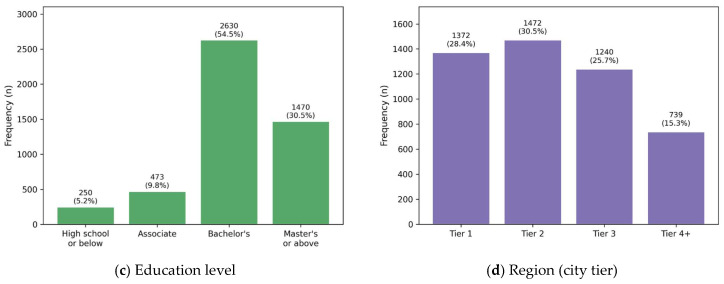
Sociodemographic profile of respondents (*N* = 4823). (**a**) Age distribution; (**b**) gender distribution; (**c**) educational attainment; (**d**) city tier.

After excluding invalid responses, a total of 4823 valid questionnaires were included in the analysis. The sample was dominated by young respondents: 63.4% were aged 18–24 years, followed by 25–34 years (11.3%) and 35 years and above (25.2%). The gender distribution was approximately balanced (female 52.4%; male 47.6%). Educational attainment was high: 54.5% held a bachelor’s degree, and 30.5% held a master’s degree or above. The distribution of city tier indicated a degree of urban concentration, with 59.0% of respondents residing in tier-1 or tier-2 cities. Full sociodemographic statistics are presented in Table 2, with visualization in Figure 1.

### 3.2. Consumption Motivations and Barriers

**Figure 2:**
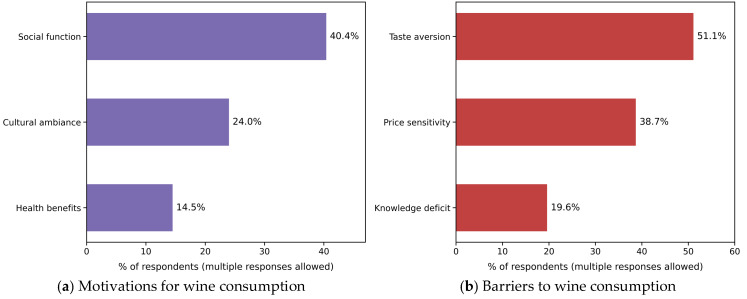
(**a**) Wine-consumption motivations and (**b**) barriers (percentages of the total sample, *N* = 4823). Multiple selections were allowed; therefore, the totals need not sum to 100%.

Overall, 67.9% of respondents held a positive attitude toward wine. With respect to consumption motivations, the most frequently endorsed were social function (40.4%), cultural ambience (24.0%), and health benefits (14.5%). With respect to consumption barriers, taste aversion was the most frequently endorsed barrier (51.1%), followed by price sensitivity (38.7%) and lack of knowledge (19.6%)—the latter referring to an inability to confidently select, evaluate, or pair wine. Respondents could select multiple motivations and multiple barriers; therefore, the frequencies within each domain need not sum to 100% (Figure 2). It should be noted that these frequencies describe only how frequently a barrier was endorsed and do not directly indicate the role that each variable plays in the broader conditional dependence structure, which is examined in Section 3.4.

### 3.3. Sociodemographic Differences in Wine Consumption

#### 3.3.1. Age and Price Tolerance

**Figure 3:**
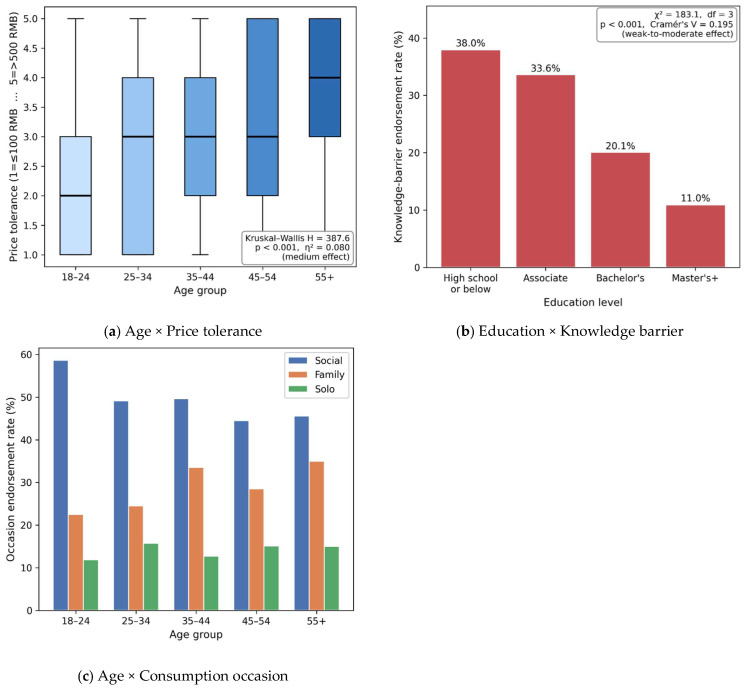
Sociodemographic differences in wine consumption. (**a**) Distribution of price tolerance by age group. (**b**) Endorsement of the knowledge barrier by educational attainment. (**c**) Endorsement rates of the three consumption occasions by age group.

The Kruskal–Wallis test indicated significant differences in price tolerance across age groups (*H*(4) = 387.6, *p* < 0.001, *η*^2^ = 0.080). This effect size corresponds to a medium effect according to Cohen’s criteria [35]. As shown in Figure 3a, price tolerance increased monotonically with age: the median for the 18–24 group was ≈ 2 (corresponding to the 101–200 CNY band); the median for the 55+ group was ≈ 4 (corresponding to the 301–500 CNY band).

#### 3.3.2. Education and the Knowledge Barrier

A chi-square test of independence indicated an association between education and endorsement of the knowledge barrier (*χ*^2^ = 183.1, *p* < 0.001, Cramér’s *V* = 0.195). According to the classification of Rea and Parker [41], this value corresponds to a weak-to-moderate association. As shown in Figure 3b, endorsement of the knowledge barrier decreased monotonically with rising educational attainment: 38.0% for high school or below, 33.6% for junior college, 20.1% for bachelor’s, and 11.0% for master’s or above.

#### 3.3.3. Gender-Related Differences

The Mann–Whitney U test detected no statistically meaningful difference in overall wine attitude between male and female respondents (*U* = 2,939,838, *p* = 0.366, rank-biserial *r* = −0.013). Both the *p*-value and the negligible effect size indicate that gender did not differentiate overall wine attitude in this young, highly educated sample. As no meaningful between-group difference emerged, no separate panel for gender is presented in Figure 3.

#### 3.3.4. Age and Consumption Occasion

As shown in Figure 3c, the three consumption occasions exhibited systematic differences in endorsement rates across age groups. Endorsement of social occasions was highest in the 18–24 group (approximately 58%) and tended to decline with age, reaching approximately 45% in the 55+ group. Endorsement of family occasions showed the opposite trend, rising monotonically from approximately 22% in the 18–24 group to approximately 35% in the 55+ group. Endorsement of solo occasions remained relatively stable across age groups, in the range of 12–16%.

### 3.4. Regularized Partial-Correlation Network

#### 3.4.1. Overall Network Structure

**Figure 4:**
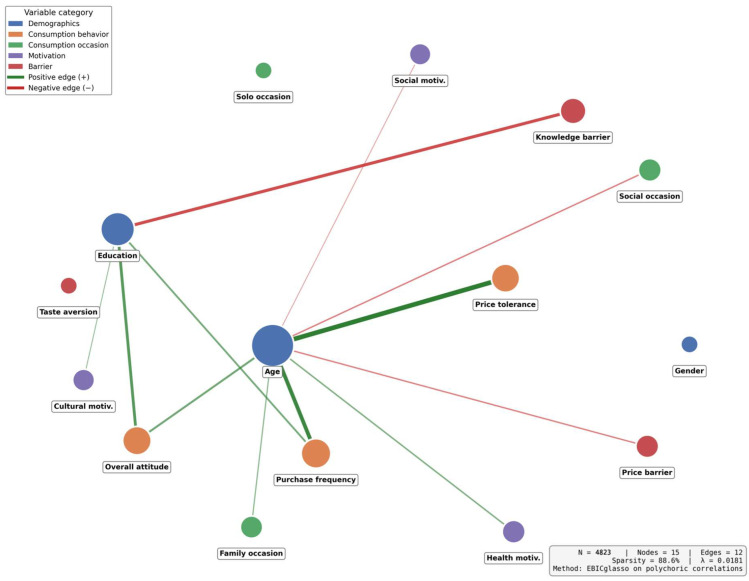
Regularized partial-correlation network (GGM) of wine consumer behavior (*N* = 4823; *λ* = 0.018; 12 of 105 possible edges retained; sparsity = 88.6%). Node colors denote variable category: blue = demographics; orange = consumption behavior; green = consumption occasion; purple = motivation; red = barrier. Node size is proportional to strength centrality. Edge thickness reflects the absolute magnitude of the partial correlation; green edges represent positive associations, and red edges represent negative associations. Isolated nodes (gender, taste aversion, and solo occasion) had no edges retained after regularization, indicating conditional independence from all other variables given the remaining network.

**Figure A1:**
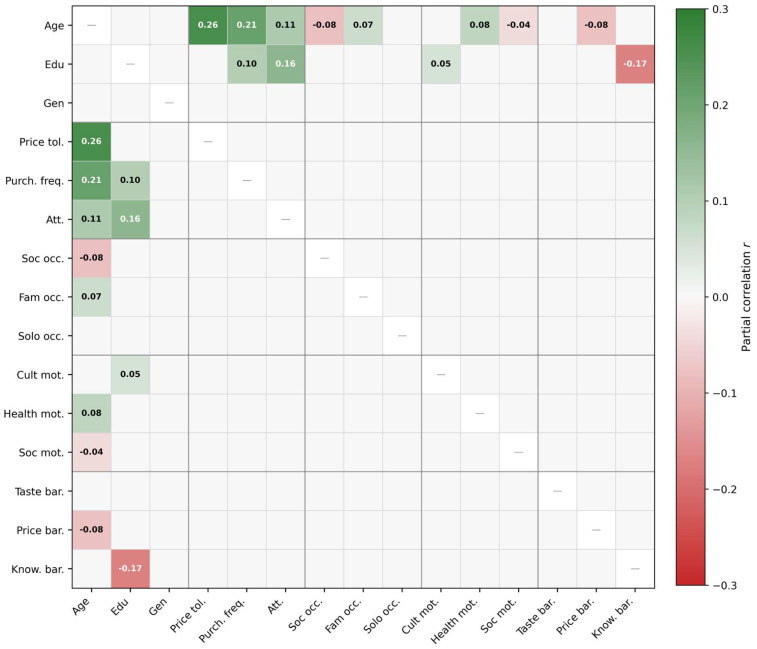
Partial-correlation matrix of the regularized Gaussian graphical model network (*N* = 4823; *λ* = 0.018; sparsity = 88.6%). Color scale: green = positive partial correlation; red = negative partial correlation. “—” indicates that the edge was removed by the EBICglasso regularization. Values are rounded to two decimal places.

A regularized partial-correlation network (GGM) was estimated on a polychoric correlation matrix using the graphical LASSO algorithm with EBIC tuning (*γ* = 0.5), yielding *λ* = 0.018. The resulting network (Figure 4) comprised 15 nodes and 12 retained edges out of 105 possible dyads (sparsity = 88.6%); the full partial-correlation matrix is provided in Appendix A (Figure A1). This level of sparsity indicates that, after conditioning on all other variables, the majority of pairwise associations were not statistically supported above the regularization threshold.

#### 3.4.2. Positive Associations

The clearest positive edge connected age and price tolerance (partial *r* ≈ +0.259), indicating that older respondents exhibited a higher willingness to pay for wine after adjustment for other variables in the network. A second positive edge linked age and purchase frequency (partial *r* ≈ +0.212). Education was positively associated with overall attitude (partial *r* ≈ +0.158) and, to a smaller degree, with purchase frequency (partial *r* ≈ +0.101). Age also showed smaller positive edges to overall attitude (partial *r* ≈ +0.112), health motivation (partial *r* ≈ +0.083), and family occasion (partial *r* ≈ +0.065); education also showed a smaller positive edge to cultural motivation (partial *r* ≈ +0.049).

#### 3.4.3. Negative Associations

The clearest negative edge in the network-connected education and the knowledge barrier (partial *r* ≈ −0.171), indicating that higher educational attainment was directly associated with a lower probability of endorsing knowledge-related barriers, even after adjustment for age and other network variables. Age also exhibited smaller negative edges to social occasion (partial *r* ≈ −0.078), price barrier (partial *r* ≈ −0.076), and social motivation (partial *r* ≈ −0.042).

#### 3.4.4. Variables Without Edges

Three variables—gender, taste aversion, and solo consumption occasion—had no edges retained after regularization. These variables were thus conditionally independent of all other variables in the network, given the remaining variable set. We note that this result does not contradict any bivariate associations that these variables may show in the prior literature; rather, any such bivariate associations may be statistically accounted for by age and education within the present network structure.

The case of taste aversion is worth highlighting given its high descriptive prevalence (51.1%; Section 3.2). The fact that the most frequently endorsed barrier exhibits no clear conditional dependency with sociodemographic, motivational, or behavioral variables represents a differential pattern of network embedding among the three consumption barriers. We interpret this pattern cautiously and discuss its implications further in Section 4.4.

### 3.5. Centrality Indices

**Figure 5:**
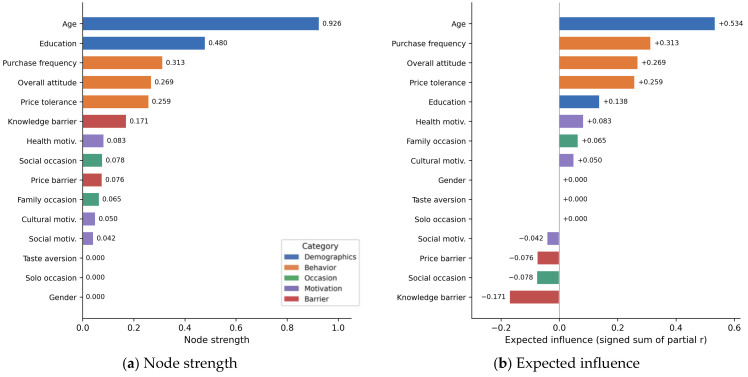
Network centrality indices. (**a**) Node strength (sum of absolute partial correlations). (**b**) Expected influence (signed sum of partial correlations).

Strength and expected influence (EI) were computed for the 15 nodes in the network (Figure 5; Table 3). Age and education emerged as the most central sociodemographic nodes (strength = 0.926 and 0.480, respectively). Among the behavioral variables, purchase frequency, overall attitude, and price tolerance formed a behaviorally central cluster (strength = 0.313, 0.269, 0.259), each with a positive expected influence. The variable with the most negative expected influence was the knowledge barrier (EI = −0.171), reflecting its inverse association with education. Following Bringmann et al. [40], closeness and betweenness centralities were not interpreted, as these metrics exhibit limited reliability in sparse regularized networks.

Expected influence (EI) provides a more interpretable index than raw strength when the signed direction of associations is meaningful. In the present network, education has a moderately high strength (0.480) but a more modest EI (+0.138), because its positive edges (to attitude, purchase frequency, and cultural motivation) are partly counterbalanced by its negative edge to the knowledge barrier.

### 3.6. Network Accuracy and Stability

Following the procedures described in Section 2.5.4, the correlation-stability coefficient was 0.59 for strength and 0.67 for expected influence, indicating high stability of the centrality estimates [37]. The bootstrapped 95% confidence intervals excluded zero for the principal edges (age–price tolerance, [0.232, 0.285]; age–purchase frequency, [0.185, 0.239]; education–knowledge barrier, [−0.198, −0.143]; education–attitude, [0.130, 0.185]), whereas several smaller edges (|partial r| < 0.10) showed wider intervals that occasionally included zero across resamples, supporting the cautious interpretation adopted for these edges.

## 4. Discussion

### 4.1. Overview of Main Findings

This study characterized the conditional dependence structure of wine-consumption behavior in young, highly educated Chinese adults. Age and education were the most central sociodemographic nodes in the network; the knowledge barrier showed a clear negative conditional association with education; taste aversion, although the most frequently endorsed barrier (51.1%), did not exhibit clear conditional dependencies with sociodemographic or motivational variables. Gender was not conditionally associated with any other variable in the network.

### 4.2. Sociodemographic Patterns of Wine Consumption

The increase in price tolerance with age observed in this study (Kruskal–Wallis *η*^2^ = 0.080, a medium effect) is broadly consistent with findings from mature Western wine markets, where older consumers (e.g., baby boomers) tend toward higher spending [1,12,14]. This pattern can also be interpreted from a life-cycle perspective: older respondents have had more exposure to wine-consumption occasions over time and tend to have higher disposable income, thereby increasing both the probability of encountering higher-priced wines and the financial capacity to purchase them [42]. From a socialization perspective, wine consumption in China remains closely tied to business banquets, family gatherings, and festive celebrations—occasions that often serve as vehicles for social connection and the display of wealth and “face” and that occur with greater frequency and ritual significance in older groups [5,26]. Taken together, this age gradient replicates, in a Chinese sample, the price-segmentation patterns documented among U.S. consumers [14] and extends them to a market in which wine adoption is comparatively recent.

The association between education and the knowledge barrier (Cramér’s *V* = 0.195) is consistent with the existing literature, which suggests that education helps consumers increase their confidence in wine knowledge and acquire the vocabulary needed to describe complex styles [17,18]. In the present sample, endorsement of the knowledge barrier declined monotonically with rising educational attainment, and this gradient persisted in the network analysis even after adjustment for age and other covariates. This confirms cross-national evidence that wine knowledge and subjective confidence rise with education [15,16] and is consistent with intervention-oriented work on wine education for Chinese consumers [18].

On the other hand, the limited gender effect observed in this study contrasts sharply with the male-dominant wine-consumption pattern commonly reported in Western adult populations [14,27,43]. Two explanations are plausible and may operate in parallel. Cohort-Specific Convergence: Young Chinese female consumers are increasingly the target audience of wine marketing campaigns that emphasize health, lifestyle, and cultural taste, which may have substantially narrowed the traditional gender gap. Sample Composition: The present sample is dominated by young and highly educated individuals and may not reflect the gender-stratified drinking norms typical of older or less educated Chinese populations. This extends to mainland China the cohort-convergence pattern documented in global epidemiological syntheses [21,22] and parallels the limited gender differentiation reported in a young, highly educated European sample [9].

### 4.3. Findings from the Network Analysis

The regularized partial-correlation network estimated in this study retained 12 of 105 possible edges (sparsity = 88.6%), a level of sparsity consistent with that typically observed when LASSO regularization is applied in psychological network analysis [37]. Furthermore, the analysis of network density suggests that, although the consumption-decision logic underlying these variables is complex, the core drivers display clear clustering, a pattern that is compatible with—though, given the small magnitudes of several of these edges, does not by itself establish—a role of knowledge and education in mitigating consumption-related anxiety [30]. These findings suggest that wine marketing strategies aimed at young, highly educated populations should shift from purely price-driven approaches toward background-aware cognitive empowerment in order to systematically reduce information asymmetry during market entry [7].

#### 4.3.1. Sociodemographic Hub Variables and Behavioral Anchors

The network exhibits a clear two-tier hub structure: Age and education (strength = 0.926 and 0.480, respectively) function as sociodemographic hub variables, with their variance linked to multiple downstream behavioral nodes; price tolerance, purchase frequency, and overall attitude form a behavioral-anchor cluster with positive expected influence, consistent with their role as facilitators of behavior. Conversely, the knowledge barrier node exhibits a negative expected influence, suggesting—tentatively, given the modest magnitude of the relevant edge (partial *r* ≈ −0.171)—that knowledge-related concerns may act as one inhibitory factor in the uptake of wine consumption [44,45].

#### 4.3.2. Education as a Bidirectional Node

A subtle finding of the present study concerns the asymmetric role of education: Education has a relatively high node strength (0.480) but a comparatively modest expected influence (+0.138), because it simultaneously presents multiple positive edges (to overall attitude, purchase frequency, and cultural motivation) and one negative edge (to the knowledge barrier). Education can thus be regarded as a bidirectional node that both promotes positive consumption behavior and suppresses a key inhibitory factor (the knowledge barrier). This dual role provides a parsimonious explanation for the often-observed pattern whereby education is identified as a predictor of wine consumption in cross-sectional surveys but with effect-size magnitudes that are inconsistent across studies—bivariate correlations capture only one dimension of a more complex conditional structure [1,5].

#### 4.3.3. Interpretation of Variables Without Edges

Variables without retained edges after regularization are conditionally independent of all other variables in the network. This result does not preclude any bivariate associations that such variables may exhibit in the prior literature [27,29,44]. Rather, within the present network structure, such bivariate associations may be statistically accounted for by age and education.

### 4.4. Differentiated Roles of the Three Consumption Barriers in the Network

A noteworthy observation is that the three consumption barriers exhibit differentiated roles in the network. Taste aversion was the most frequently endorsed barrier (51.1%), yet it had no edges in the regularized network, appearing as a structurally isolated node [7]. The knowledge barrier, despite a lower endorsement rate (19.6%), showed a clear negative conditional association with education (partial *r* ≈ −0.171) [18,44]. The price barrier (38.7%) fell between these two, exhibiting weaker edges. Descriptively, taste aversion was relatively evenly distributed across sociodemographic strata such as age, gender, and education, whereas knowledge concerns were concentrated mainly in groups with lower educational attainment [5,45].

We interpret this pattern with caution. The dissociation between frequency and conditional centrality is compatible with the possibility that taste aversion operates as a population-level sensory barrier, whereas lack of knowledge operates through a sociodemographic gradient—a distinction that, if replicated, would have different implications for intervention design [29]. However, alternative explanations are equally plausible: The isolation of taste aversion may reflect measurement properties of a single binary item rather than a substantively distinct mechanism; the association between education and the knowledge barrier may partly reflect shared variance with unmeasured cultural capital variables [18]; cross-sectional data cannot rule out reverse or bidirectional causal pathways [44]. We therefore regard these observations as exploratory rather than confirmatory and encourage subsequent longitudinal and intervention studies to test whether the differentiated roles described here can be replicated and translated into practical intervention efficacy. The structural isolation of taste aversion despite its high prevalence echoes qualitative accounts of wine avoiders, for whom sensory dislike is primary and largely undifferentiated across social strata [29], and is consistent with sensory studies showing that Chinese consumers’ liking is driven strongly by sweetness and fruit character [46].

### 4.5. Limitations

Several limitations of this study should be made explicit. First, the cross-sectional design does not support causal inference. The conditional associations identified in the network represent statistical patterns at a single point in time and cannot establish temporal precedence or rule out unmeasured common causes. Second, the sample was recruited through non-probability snowball sampling and is heavily skewed toward respondents aged 18–24 years (63.4%) and toward holders of tertiary degrees (85.0% bachelor’s degree or above). Although this skew partly reflects a deliberate focus on the fastest-growing segment of the Chinese wine market [1,45], the findings characterize a predominantly young, highly educated, and urban subgroup and cannot be generalized to the population of Chinese adults or of Chinese wine consumers as a whole; in particular, the descriptive prevalence estimates (e.g., the 67.9% positive attitude toward wine) are properties of this convenience sample and should not be interpreted as population parameters. Third, part of the sample was recruited at wine retail stores, tasting events, and restaurants. Such venue-based recruitment plausibly over-represents consumers who are already interested in, and knowledgeable about, wine; descriptive endorsement rates—most notably the proportion holding a positive attitude, the reported purchase frequencies, and the endorsement of consumption motivations—are therefore best interpreted as upper-bound estimates for the accessible population, and an influence of the recruitment strategy on the estimated dependence structure itself cannot be excluded. Fourth, the instrument was not adapted from a previously validated psychometric scale, no test–retest data were collected, and its single-item behavioral indicators cannot be checked for internal consistency; item construction was, however, informed by established frameworks [1], reviewed by industry and academic experts, and examined in a pilot study (n = 52), and conclusions at the level of individual constructs should be interpreted with corresponding caution. Fifth, several variables known to relate to wine consumption—household income, occupational status, marital status, overseas experience, and interest in Western culture—were not measured. This reflects a deliberate design trade-off: the instrument was kept very brief (median completion time ≈ 3 min) to sustain data quality across heterogeneous recruitment channels; because a partial-correlation network is conditional on the variables included in it, the omission of these factors may simplify the estimated structure, and individual edges could change if the variable set were extended [32,33]. Sixth, three variables were excluded from the network model for methodological and conceptual reasons detailed in Section 2.4; this boundary specification decision should be borne in mind when comparing the present network with studies that include macro-level or geographic variables. In particular, regional and urbanization effects were not modeled at the individual level in this study.

### 4.6. Future Research Directions

Building on the present findings, we propose seven priority directions for future research. First, longitudinal research—following the same respondents for 3–5 years—should be carried out to directly test whether the conditional associations observed here are maintained, attenuated, or transformed as the cohort ages [47]. Second, cross-cultural replication should be considered, testing the present conclusions in parallel samples from China, Japan, and Korea to determine whether the differentiated barrier pattern is generalizable across emerging East Asian wine markets or culturally specific [48,49]. Third, sensory experimental research should be performed—directly manipulating wine styles to test whether product-level interventions can alleviate the taste-aversion barrier [50,51]. Fourth, intervention studies are recommended—deploying simplified labels and sommelier-guided retail experiences in consumer groups with lower educational attainment to test the causal claims implicit in the present interpretation of the knowledge barrier [18,50]. Fifth, mixed-methods follow-up studies should be carried out—using qualitative interviews with taste-averse consumers to gain a deeper understanding of the phenomenological content underlying the statistical patterns observed here [29]. Sixth, examining regional and urbanization effects would be valuable, using designs better suited to macro-level variables, such as multilevel models [9]. Seventh, future surveys should incorporate household income, occupational status, marital status, overseas experience, and interest in Western culture as covariates, both to test the robustness of the edges reported here and to extend the boundary of the estimated network [32,33].

### 4.7. Practical and Policy Implications

For wine producers, importers, and marketers operating in the Chinese market, the present findings suggest a dual-track strategic perspective [7]. The product track could prioritize sensory friendliness—developing entry-level wines with more accessible profiles (e.g., styles emphasizing red fruit, sweetness, and fruit-driven finishes)—in an effort to address taste-related reluctance, the most frequently reported barrier in this sample [29,46,52]—while noting that, because this variable was structurally isolated in the network, the expected leverage of such product-level interventions on other consumption variables remains an open empirical question. The education track could prioritize lowering the knowledge barrier in consumer groups with lower educational attainment through simplified labeling, sommelier-guided retail experiences, and partnerships with online education platforms [7,18]. Because education functions in the network both as a sociodemographic hub variable with positive downstream edges and as a node with a negative edge to the knowledge barrier, interventions that can functionally substitute for formal educational capital may simultaneously affect multiple downstream variables through the network [30].

For Chinese policymakers and regulators, the present findings also suggest that culturally sensitive consumer-education initiatives could serve as a useful complement to market-development planning—an approach analogous to the information actions in EU wine policy aimed at protecting geographic-indication wines and encouraging responsible consumption [53]. The findings concerning the knowledge barrier in particular suggest that the existing higher-education infrastructure could be considered as a channel for disseminating basic wine knowledge to address the genuine consumer knowledge gap, without resorting to excessive commercial promotion.

## 5. Conclusions

This study used a regularized Gaussian graphical model (GGM) to characterize the conditional dependence structure of wine-consumption behavior in 4823 young, highly educated Chinese adults. Age and education were the most important sociodemographic nodes in the consumer-behavior network—age was associated with higher price tolerance and purchase frequency, whereas education was associated with a more positive consumption attitude and a lower probability of endorsing the knowledge barrier (partial *r* ≈ −0.171). A noteworthy exploratory observation concerns the differentiated network embedding of the three consumption barriers: Taste aversion, although the most frequently endorsed barrier (51.1%), did not show clear conditional dependencies with sociodemographic or motivational variables, whereas the knowledge barrier, despite exhibiting a lower endorsement rate (19.6%), showed a clearer gradient with education. The price barrier (38.7%) occupied an intermediate position, showing weaker network edges. This pattern, if validated in future research, suggests that the three consumption barriers may operate through different pathways: Sensory-level concerns may require product-level interventions, whereas knowledge-level concerns may be more amenable to targeted education and simplified labeling. Gender was not conditionally associated with any other variable in the network, suggesting that traditional gender-stratified consumption patterns have weakened substantially in this study population. These findings offer a perspective on market development for emerging Chinese wine consumer segments that complements status-driven luxury positioning with two additional dimensions: sensory friendliness and knowledge accessibility. Longitudinal, cross-cultural, and intervention research is needed to verify whether these patterns are replicable in other East Asian markets and to translate them into quantifiable intervention effects.

## Figures and Tables

**Table 1:** Variable operationalization and network inclusion.

Item	Variable	Construct	Type	Coding	In Network?
Q1	age_group	Age	Ordinal	1–5	Yes
Q2	gender	Gender	Binary	0, 1	Yes
Q3	education	Education	Ordinal	1–4	Yes
Q4	region	City tier	Ordinal	1–4	No ^a^
Q5	attitude	Overall attitude	Ordinal	1–3	Yes
Q6	price_tolerance	Price tolerance	Ordinal	1–5	Yes
Q7	purchase_freq	Purchase frequency	Ordinal	1–4	Yes
Q8a	scene_social	Social occasion	Binary	0, 1	Yes
Q8b	scene_family	Family occasion	Binary	0, 1	Yes
Q8c	scene_alone	Solo occasion	Binary	0, 1	Yes
Q8d	scene_business	Business occasion	Binary	0, 1	No ^b^
Q9a	attract_culture	Cultural motivation	Binary	0, 1	Yes
Q9b	attract_health	Health motivation	Binary	0, 1	Yes
Q9c	attract_social	Social motivation	Binary	0, 1	Yes
Q9d	attract_taste	Taste preference	Binary	0, 1	No ^c^
Q10a	barrier_taste	Taste aversion	Binary	0, 1	Yes
Q10b	barrier_price	Price barrier	Binary	0, 1	Yes
Q10c	barrier_knowledge	Knowledge barrier	Binary	0, 1	Yes

Notes: ^a^ Geographic location was recorded as a nominal variable (province/city) and was therefore not eligible for inclusion in the network, which requires ordinal or binary inputs (see Section 2.4); it was retained for sample description only. ^b^ Excluded because of low-variance constraints (endorsement rate < 5%) [32]. ^c^ Excluded to avoid synonymous association with barrier_taste (Q10a).

**Table 2:** Sample sociodemographic characteristics (*N* = 4823).

Variable	Category	*N*	%
Age	18–24 years	3060	63.4
	25–34 years	547	11.3
	35–44 years	552	11.4
	45–54 years	418	8.7
	55 years and above	246	5.1
Gender	Male	2295	47.6
	Female	2528	52.4
Education	High school or below	250	5.2
	Junior college	473	9.8
	Bachelor’s	2630	54.5
	Master’s or above	1470	30.5
City tier	Tier 1	1372	28.4
	Tier 2	1472	30.5
	Tier 3	1240	25.7
	Tier 4 or below	739	15.3

**Table 3:** Network centrality indices (in descending order of strength).

Rank	Node	Category	Strength	Expected Influence
1	Age	Demographic	0.926	+0.534
2	Education	Demographic	0.480	+0.138
3	Purchase frequency	Behavior	0.313	+0.313
4	Overall attitude	Behavior	0.269	+0.269
5	Price tolerance	Behavior	0.259	+0.259
6	Knowledge barrier	Barrier	0.171	−0.171
7	Health motivation	Motivation	0.083	+0.083
8	Social occasion	Occasion	0.078	−0.078
9	Price barrier	Barrier	0.076	−0.076
10	Family occasion	Occasion	0.065	+0.065
11	Cultural motivation	Motivation	0.049	+0.049
12	Social motivation	Motivation	0.042	−0.042
13–15	Solo occasion/Taste aversion/Gender	Occasion/Barrier/Demographic	0.000	0.000

Notes: Variables that are conditionally independent of all other variables (no retained edges after regularization) are listed in the last row. Closeness and betweenness centralities are not reported because of their limited reliability in sparse regularized networks [40].

## Data Availability

The data presented in this study are available on request from the corresponding authors due to privacy and ethical restrictions.

## References

[1] Lockshin L., Corsi A.M. (2012). Consumer behaviour for wine 2.0: A review since 2003 and future directions. Wine Econ. Policy.

[2] Monteiro Vieira I.M., Passos Santos B.L., Santos Ruzene D., Brányik T., Teixeira J.A., de Almeida Silva J.B.E., Pereira Silva D. (2018). Alcohol and Health: Standards of Consumption, Benefits and Harm—A Review. Czech. J. Food Sci..

[3] Del Rey R., Loose S. (2023). State of the International Wine Market in 2022: New market trends for wines require new strategies. Wine Econ. Policy.

[4] Ohana-Levi N., Netzer Y. (2023). Long-Term Trends of Global Wine Market. Agriculture.

[5] García-Cortijo M.C., Villanueva E.C., Castillo-Valero J.S., Li Y. (2019). Wine consumption in China: Profiling the 21st century Chinese wine consumer. Ciênc. Téc. Vitiviníc..

[6] Mackenzie M., Weber K., Fountain J., Abbasi R. (2024). Segmenting Chinese wine consumers on the basis of wine knowledge and consumption behavior. Int. J. Wine Bus. Res..

[7] Camillo A.A. (2012). A strategic investigation of the determinants of wine consumption in China. Int. J. Wine Bus. Res..

[8] Masset P., Weisskopf J.P., Faye B., Le Fur E. (2016). Red obsession: The ascent of fine wine in China. Emerg. Mark. Rev..

[9] Sandri E., Capoferri M., Luciani G., Piredda M. (2025). Alcohol Consumption and Beverage Preferences in a Predominantly Female, Highly Educated Spanish Population: A Sociodemographic and Network Analysis. Foods.

[10] Yu Y., Sun H., Goodman S., Chen S., Ma H. (2009). Chinese choices: A survey of wine consumers in Beijing. Int. J. Wine Bus. Res..

[11] Bruwer J., Saliba A., Miller B. (2011). Consumer behaviour and sensory preference differences: Implications for wine product marketing. J. Consum. Mark..

[12] Wolf M.M., Higgins L.M., Wolf M.J., Qenani E. (2018). Do generations matter for wine segmentation?. J. Wine Res..

[13] Olsen J.E., Thach And L., Nowak L. (2007). Wine for My Generation: Exploring How US Wine Consumers are Socialized to Wine. J. Wine Res..

[14] Thach L., Olsen J. (2015). Profiling the high frequency wine consumer by price segmentation in the US market. Wine Econ. Policy.

[15] Forbes S., Cohen D., Dean D. (2008). An Assessment of Wine Knowledge Amongst Global Consumers. Lincoln University Research Archive. https://hdl.handle.net/10182/3405.

[16] Johnson T.E., Bastian S.E.P. (2007). A preliminary study of the relationship between Australian wine consumers’ wine expertise and their wine purchasing and consumption behaviour. Aust. J. Grape Wine Res..

[17] Latour K.A., Latour M.S. (2010). Bridging Aficionados’ Perceptual and Conceptual Knowledge to Enhance How They Learn from Experience. J. Consum. Res..

[18] Pelet J.E., Canziani B., Terblanche N. (2024). Adapting online wine education to China: A two-study multimethod approach. Int. J. Wine Bus. Res..

[19] Kerr-Corrêa F., Igami T.Z., Hiroce V., Tucci A.M. (2007). Patterns of alcohol use between genders: A cross-cultural evaluation. J. Affect. Disord..

[20] Wilsnack R.W., Wilsnack S.C., Kristjanson A.F., Vogeltanz-Holm N.D., Gmel G. (2009). Gender and alcohol consumption: Patterns from the multinational GENACIS project. Addiction.

[21] Keyes K.M. (2021). Age, Period, and Cohort Effects in Alcohol Use in the United States in the 20th and 21st Centuries. Alcohol Res. Curr. Rev..

[22] Slade T., Chapman C., Swift W., Keyes K., Tonks Z., Teesson M. (2016). Birth cohort trends in the global epidemiology of alcohol use and alcohol-related harms in men and women: Systematic review and metaregression. BMJ Open.

[23] Hall J., Binney W., Barry O’Mahony G. (2004). Age Related Motivational Segmentation of Wine Consumption in a Hospitality Setting. Int. J. Wine Mark..

[24] Bruwer J., Li E. (2007). Wine-Related Lifestyle (WRL) Market Segmentation: Demographic and Behavioural Factors. J. Wine Res..

[25] Peng D., Huang C.H. (2024). Exploring the hierarchy of motivations in the wine purchase behavior of Chinese young wine consumers. Int. J. Wine Bus. Res..

[26] Liu F., Murphy J. (2007). A qualitative study of Chinese wine consumption and purchasing. Int. J. Wine Bus. Res..

[27] Thach L. (2012). Time for wine? Identifying differences in wine-drinking occasions for male and female wine consumers. J. Wine Res..

[28] Lesschaeve I., Noble A.C. (2005). Polyphenols: Factors influencing their sensory properties and their effects on food and beverage preferences. Am. J. Clin. Nutr..

[29] McIntyre E., Ovington L.A., Saliba A.J., Moran C.C. (2015). Qualitative study of alcohol consumers who choose to avoid wine. Aust. J. Grape Wine Res..

[30] Hussain M., Cholette S., Castaldi R. (2007). Determinants of wine consumption of US consumers: An econometric analysis. Int. J. Wine Bus. Res..

[31] Yabin W., Li J. (2019). Segmentation of China’s online wine market based on the wine-related lifestyle. Br. Food J..

[32] Epskamp S., Fried E.I. (2018). A tutorial on regularized partial correlation networks. Psychol. Methods.

[33] Borsboom D., Cramer A.O.J. (2013). Network Analysis: An Integrative Approach to the Structure of Psychopathology. Annu. Rev. Clin. Psychol..

[34] Meade A.W., Craig S.B. (2012). Identifying careless responses in survey data. Psychol. Methods.

[35] Lachenbruch P.A., Cohen J. (1989). Statistical Power Analysis for the Behavioral Sciences (2nd ed.). J. Am. Stat. Assoc..

[36] Foygel R., Drton M. (2010). Extended Bayesian Information Criteria for Gaussian Graphical Models. arXiv.

[37] Epskamp S., Borsboom D., Fried E.I. (2017). Estimating psychological networks and their accuracy: A tutorial paper. Behav. Res..

[38] Isvoranu A.-M., Epskamp S. (2023). Which estimation method to choose in network psychometrics? Deriving guidelines for applied researchers. Psychol. Methods.

[39] Robinaugh D.J., Millner A.J., McNally R.J. (2016). Identifying highly influential nodes in the complicated grief network. J. Abnorm. Psychol..

[40] Bringmann L.F., Elmer T., Epskamp S., Krause R.W., Schoch D., Wichers M., Wigman J.T., Snippe E. (2019). What do centrality measures measure in psychological networks?. J. Abnorm. Psychol..

[41] Rea L.M., Parker R.A. (2014). Designing and Conducting Survey Research: A Comprehensive Guide.

[42] Ferreira C., Rebelo J., Lourenço-Gomes L., Correia E., Baumert P., Plumejeaud C. (2020). Consumer preferences and purchasing rationales for wine: A multivariate data analysis. New Medit..

[43] Rodríguez-Donate M.C., Romero-Rodríguez M.E., Cano-Fernández V.J., Guirao-Pérez G. (2019). Gender and wine consumption: Sociodemographic profiles. Br. Food J..

[44] Li J., Jia J., Taylor D., Bruwer J., Li E. (2011). The wine drinking behaviour of young adults: An exploratory study in China. Br. Food J..

[45] Yap M.H.T., Chen N. (2017). Understanding young Chinese wine consumers through innovation diffusion theory. Tour. Hosp. Manag..

[46] Williamson P.O., Robichaud J., Francis I.L. (2012). Comparison of Chinese and Australian consumers’ liking responses for red wines. Aust. J. Grape Wine Res..

[47] Shi F., Ji S., Weaver D., Huang M.F. (2021). From curious to connoisseur: A longitudinal segmentation of attendees at a Chinese wine festival. Int. J. Contemp. Hosp. Manag..

[48] Corsi A.M., Marinelli N., Alampi Sottini V. (2013). Italian wines and Asia: Policy scenarios and competitive dynamics. Br. Food J..

[49] Rod M., Beal T. (2014). The experience of New Zealand in the evolving wine markets of Japan and Singapore. Asia-Pac. J. Bus. Adm..

[50] Luo A., Quadri-Felitti D.L., Mattila A.S. (2024). The double-edged effects of visualizing wine style: Sweetness scale on wine label. Int. J. Contemp. Hosp. Manag..

[51] Saltman Y., Johnson T.E., Wilkinson K.L., Ristic R., Norris L.M., Bastian S.E.P. (2017). Natural Flavor Additives Influence the Sensory Perception and Consumer Liking of Australian Chardonnay and Shiraz Wines. Am. J. Enol. Vitic..

[52] Chu X., Li Y., Xie Y., Tian D., Mu W. (2019). Regional difference analyzing and prediction model building for Chinese wine consumers’ sensory preference. Br. Food J..

[53] Pomarici E., Sardone R. (2020). EU wine policy in the framework of the CAP: Post-2020 challenges. Agric. Econ..

